# Subthalamic deep brain stimulation improves smooth pursuit and saccade performance in patients with Parkinson’s disease

**DOI:** 10.1186/1743-0003-10-33

**Published:** 2013-04-03

**Authors:** Maria H Nilsson, Mitesh Patel, Stig Rehncrona, Måns Magnusson, Per-Anders Fransson

**Affiliations:** 1 Department of Health Sciences, Lund University, Lund, Sweden; 2 Department of Neurosurgery, Clinical Sciences Lund, Skåne University Hospital, Lund, Sweden; 3 Department of Clinical Sciences, Lund University, Lund S-221 85, Sweden; 4 Department of Clinical Neuroscience, Neuro-Otology Department, Imperial College London, London, UK

**Keywords:** Parkinson’s disease, Deep brain stimulation, Subthalamic nucleus, Oculomotor functions

## Abstract

**Background:**

Deep brain stimulation (DBS) in the subthalamic nucleus (STN) significantly reduces symptoms of Parkinson’s disease (PD) such as bradykinesia, tremor and rigidity. It also reduces the need for anti-PD medication, and thereby potential side-effects of _L_-Dopa. Although DBS in the STN is a highly effective therapeutic intervention in PD, its mechanism and effects on oculomotor eye movement control and particularly smooth pursuit eye movements have to date rarely been investigated. Furthermore, previous reports provide conflicting information. The aim was to investigate how DBS in STN affected oculomotor performance in persons with PD using novel analysis techniques.

**Methods:**

Twenty-five patients were eligible (22 males, 3 females) according to the clinical inclusion criteria: idiopathic PD responsive to _L_-Dopa and having had bilateral STN stimulation for at least one year to ensure stable DBS treatment. Fifteen patients were excluded due to the strict inclusion criteria applied to avoid interacting and confounding factors when determining the effects of DBS applied alone without PD medication. One patient declined participation. Nine PD patients (median age 63, range 59–69 years) were assessed after having their PD medications withdrawn overnight. They were examined with DBS ON and OFF, with the ON/OFF order individually randomized.

**Results:**

DBS ON increased smooth pursuit velocity accuracy (p < 0.001) and smooth pursuit gain (p = 0.005), especially for faster smooth pursuits (p = 0.034). DBS ON generally increased saccade amplitude accuracy (p = 0.007) and tended to increase peak saccade velocity also (p = 0.087), specifically both saccade velocity and amplitude accuracy for the 20 and 40 degree saccades (p < 0.05). Smooth pursuit latency tended to be longer (p = 0.090) approaching normal with DBS ON. Saccade latency was unaffected.

**Conclusions:**

STN stimulation from DBS alone significantly improved both smooth pursuit and saccade performance in patients with PD. The STN stimulation enhancement found for oculomotor performance suggests clear positive implications for patients’ ability to perform tasks that rely on visual motor control and visual feedback. The new oculomotor analysis methods provide a sensitive vehicle to detect subtle pathological modifications from PD and the functional enhancements produced by STN stimulation from DBS alone.

## Background

Parkinson’s disease (PD) is the second-most common neurodegenerative disorder after Alzheimer’s disease and inflicts an increasing social and economic burden on society as population ages [1]. The prevalence of PD in industrialized countries is estimated to be about 0.3% for the entire population, 1% in people over 60 years of age [2] rising to 4% in people over 80 years of age [3].

In PD, an insufficient formation and action of dopamine in the substantia nigra pars compacta causes defective transmission of impulses from the Basal ganglia (BG) [4,5]. The BG influences many cortical functions through several parallel Basal ganglia-thalamo-cortical loops, e.g. limbic, motor and oculomotor loops [6]. For motor and oculomotor circuits, the BG is believed to play a role in selecting which initiating drives are allowed to be expressed as responses through the control of the thalamo-cortical and brainstem motor networks [7]. One of the main manifestations of PD is therefore motor dysfunction, and this forms part of its clinical diagnosis.

A diagnosis of PD is based on the presence of at least two of the following symptoms: resting tremor, bradykinesia, rigidity or postural imbalance [5]. The diagnosis is confirmed by a positive response to levodopa (_L_-Dopa) [1]. However, the degree of the motor symptoms might not be strictly related to the degree of disease progression [8,9]. Only some patients may present postural tremor; patients with striatal deformities tend to be younger and postural instability due to the loss of postural reflexes generally occurs in the later stages of PD and usually after the onset of other clinical features [5,10]. Oculomotor performance such as saccadic and smooth pursuit eye movements is also affected by PD [11-15], and this could potentially offer an additional opportunity for evaluating the disease severity or progression.

Ocular movements, including saccadic and smooth pursuit, have increasingly been used to detect subtle pathological modifications caused by neurological deficits or lesions [14,16,17]. Saccadic eye movements are high-velocity, ballistic changes in eye position that bring an object of interest onto the fovea centralis retinae, whereas smooth pursuit eye movements are tracking movements ensuring that the image of a moving object is maintained on the fovea [18]. Oculomotor responses are vital for safe movement control and orientation and therefore for many daily activities [19]. Several areas are involved in the generation of oculomotor responses such as the frontal eye field, the supplementary eye field, the dorsolateral prefrontal cortex and the superior colliculus [20,21]. These areas all receive input integrated through the BG and are regulated by inhibitory mechanisms [5].

Deep brain stimulation (DBS) in the subthalamic nucleus (STN) significantly reduces the PD symptoms of bradykinesia, tremor, rigidity and the need for anti-PD medication, which further reduces the motor complications from dopamine therapy [3]. Previous studies have shown that DBS in the STN can improve saccadic performance and orienting gaze movements [22-28]. To the best of our knowledge, only one previous study, by Pinkardt and colleagues has evaluated the specific effects of DBS on smooth pursuit [29]. This recent study assessed the effects of DBS in the STN while participants remained on anti-PD medication and showed no difference between DBS ON and DBS OFF. We intend to assess oculomotor control with DBS in the STN following withdrawal of anti-PD medication from the previous night to illustrate the effects without medication and to compare saccade and smooth pursuits on the same patients. Hence, the effect of DBS in the STN on smooth pursuit eye movements is largely unknown, whereas the described effects of PD medication on smooth pursuit performance differ between reports [11,14,30,31].

The aim of the present study was to evaluate the effect of STN stimulation alone (i.e. without anti-PD medication) on oculomotor performance in persons with PD using both traditional quantitative methods and two newer analysis methods specifically looking at smooth pursuit velocity accuracy and the ratio between saccade velocity and saccade amplitude, the latter commonly called main sequence. These newer methods have shown better sensitivity in detecting subtle pathological dysfunctions compared to conventional methods [32,33]. Therefore, these methods might further elucidate how PD influences smooth pursuit and saccade eye movements and the effectiveness of STN stimulation to enhance oculomotor functions in patients with PD. A second aim was to compare the objective recordings of ocular movements and subjective scores from the Unified Parkinson’s Disease Rating Scale (UPDRS) part III, i.e., motor examination.

## Materials and methods

### Patients

Twenty-five patients were eligible (22 men, three women) according to the inclusion criteria: idiopathic PD responsive to _L_-Dopa, between 50–70 years old and having had bilateral STN stimulation for at least one year to ensure stable DBS treatment. Fifteen patients were excluded due to the following exclusion criteria: concomitant diseases interfering with testing, an inability to cooperate or an inability to stand for two minutes without support since standing assessment is a part of the UPDRS part III. One patient declined participation.

Nine patients with PD participated in the study (median age 63, range 59–69 years). Descriptive information (e.g. L-dopa equivalents and DBS parameter settings) is provided in Table 1. The neurosurgical procedure has been described elsewhere [34]. All patients were recruited from the Department of Neurosurgery, Skåne University Hospital.

**Table 1:** Patients’ characteristics and scores of the UPDRS part III

**Sex**	**8 men, 1 woman**
Age (years) at surgery, mean (SD)	63.2 (3.9)
PD-duration (years), mean (SD)	16.3 (3.6)
L-dopa equivalents (mg/day), mean (SD)	481.5 (242.1)
Months since DBS-surgery, mean (SD)	42.0 (20.1)
DBS parameter settings, mean (SD), Amplitude (V), pulse width (μs), Frequency (Hz)	Right: 3.4 (0.54) V, 66.7 (13.2)μs, 138.3 (40.2) Hz
	Left: 3.4 (0.62) V, 66.7 (13.2)μs, 138.3 (40.2) Hz
Location of contacts with negative polarity, in relation to the midpoint of the intercommissural line (IC), mean (SD)	Right: 11.3 (0.91) mm lateral, 3.5 (0.33) mm posterior, 2.4 (1.4) mm inferior.
	Left: 11.3 (0.95) mm lateral, 3.9 (0.70) mm posterior, 2.8 (1.0) mm inferior.
	IC = 24.8 (0.59) mm
UPDRS part III scores, without anti-PD medication	
- DBS turned OFF, median (q1-q3)	42.5 (38.3-56.5)
- DBS turned ON, median (q1-q3)	22.0 (17.5-25.3)

Levodopa equivalents are calculated according to Østergaard et al., and Calne.

UPDRS part III: Unified Parkinson’s Disease Rating Scale, motor examination. Higher scores denote more severe motor symptoms. The maximum total score on the UPDRS part III is 108 points, and higher scores reflect more severe motor symptoms.

Without medication: Overnight withdrawal of all anti-Parkinsonian medication for 10–12 hours. All individuals were on L-dopa, and seven out of the nine participants were also on dopamine agonists (ranging from 20-50% of LED). When tested, all participants experienced off symptoms.

Of note, the assessments of severity of PD using UPDRS scores were done at the same occasion as the assessments of oculomotor functions.

A routine clinical neurological examination was performed six months prior to the study in all nine patients, and if needed the DBS and/or medication was adjusted to optimize treatment. The experiments were performed in accordance with the most recent Helsinki declaration. The Regional Ethical Board approved the study and all patients gave written informed consent.

#### Procedure & assessments

To investigate the effects of STN stimulation alone, all anti-PD medications were withdrawn overnight (from 10 pm) while all participants were kept as in-patients. The following morning, an independent person programmed the DBS to either ON or OFF. The order (i.e., DBS ON/OFF) was randomized to avoid any systematic differences and bias. The assessments were performed thirty minutes after programming the DBS. Short breaks were allowed between the individual tests if needed.

UPDRS part III (motor examination) was used to describe and evaluate motor symptoms [35] (Table 1). Each patient was always assessed by the same examiner i.e., a PD nurse or a neurologist. The UPDRS investigator was blind to the randomization order. The UPDRS assessment was followed by the assessments of oculomotor functions, where one test session took at its most 30 minutes. The DBS was then reprogrammed by an independent person. During the following 30 minutes, participants had a break and a light meal (fruit, sandwich and mineral water). The test session was repeated in the other DBS state and the UPDRS and oculomotor assessments performed in the same order.

#### Equipment

The visual target used in the oculomotor tests was a circular red dot with a diameter of 3mm projected onto a dark canvas screen (2m height vs. 3m width) about 1.3m in front of the subjects. The visual target was produced by a diode laser contained within a mobile over-head console, allowing optimal vertical positioning of the visual target for each individual. The eye movements were recorded by electronystagmography (ENG) by means of a bipolar recording technique. Two Ag/AgCl ENG-electrodes were placed about 1cm from the outer canthi of the eyes to measure horizontal eye movements. Two other electrodes were fixed above and below the left eye to measure the vertical eye movements and eye blinks. Finally, one ground electrode was attached on the mid-forehead. Before each test, a calibration procedure was performed to ensure that the electrical ENG signals within the range of ±30 degree amplitude in horizontal direction correctly corresponded to right and left eye movements, with an error less than 1 degree at the 30 degree amplitude. In the vertical direction, the calibration amplitude was set with reference to eye blinking. A customized program Vestcon™ controlled the visual target projection, calibration and sampled the ENG data at 200 Hz. The computer program also analyzed the ENG data.

#### Smooth pursuit eye movement recordings

All participants were tested in a completely dark room, seated in an inclined chair directly in front of a black canvas screen. They were instructed to fixate on the target and follow its movement as accurately as possible without turning their head or moving their eyes before the target movement. A custom-made headrest prevented inappropriate head movements. Fixed smooth pursuit tests sequences were used in this study to ensure that all test conditions and the test sequences themselves were identical and equally difficult to perform with DBS OFF and DBS ON. The smooth pursuit target moved horizontally with a constant velocity from side to side, with a range of ±30° of the visual field, i.e. a distance of 60° between (+) 30° to the right and (−) 30° to the left. The smooth pursuit test was performed in a set-order of different velocities: 10, 20, 30, 40, 40, 30, 20, 10°/s. At each velocity, the smooth pursuit eye movements were tested 4 times, i.e. two times per direction (right to left and left to right). When the visual target reached the maximum amplitude, either to the right or left, the position was maintained for 1 second before the next movement commenced in the opposite direction. In total, 8 smooth pursuits for each smooth pursuit target velocity investigated were analyzed, giving a total of 32 smooth pursuits analyzed for each DBS state and a sum of 64 smooth pursuits analyzed for each PD participant.

#### Saccadic eye movement recordings

The conditions and calibration before the saccade tests were identical to the smooth pursuit assessments and instructions were the same. Fixed saccade test sequences were used to ensure that all test conditions were identical and the tests equally difficult to perform with DBS OFF and DBS ON. The visual target jumped stepwise horizontally according to a sequence of different amplitudes: ±10, ±20, ±30°, yielding saccades of a total range of respectively 20, 40 and 60° amplitude. The visual target remained at each position for 1.5s seconds. The saccades were tested 10 times at each of the amplitudes, five times for saccades from right to left, and five times for saccades from left to right. The first and last saccade in each amplitude block of saccades was removed prior to analysis. In total, 8 saccades for each target amplitude investigated were analyzed, giving a total of 24 saccades analyzed for each DBS state and a sum of 48 saccades analyzed for each PD participant.

#### Smooth pursuit data analysis

The smooth pursuit analysis method has been described in detail elsewhere [32,33]. In brief, the smooth pursuit latency was defined as the time between start of target movement until the velocity of the recoded eye movement exceeded 5°/s. The calculated latency was regarded incorrect and rejected if the latency was shorter than 0.1 seconds or longer than 0.6 seconds. Smooth pursuit gain was calculated by first identifying and removing time periods where the measured eye movements were presumed to be saccades or deemed too slow to represent a smooth pursuit eye movements. Average velocity was calculated for the remaining periods, and the gain value was obtained by dividing the average eye movement velocity by the target velocity value. Smooth pursuit velocity accuracy was obtained by calculating the percentage of time during the entire movement range when the eye movement velocity was within the target velocity boundaries of less than 20% absolute error from the visual target velocity.

#### Saccadic data analysis

The saccade analysis method has been described in detail elsewhere [32,33]. In brief, prior to the analysis of the saccadic data, the recorded ENG data was low-pass filtered at a cut-off frequency of 70 Hz. Thereafter, the data was deemed to obtain the velocity of the eye movements during each individual target movement. The recorded saccade latency was defined as the time between start of target movement until the recorded eye movement velocity exceeded 80°/s. The calculated latency was regarded incorrect and rejected if the latency was below 0.1 seconds or above 0.6 seconds. The saccade was also rejected if the duration of the saccade was shorter than 25 ms.

Peak saccade velocity was calculated by first identifying and removing time periods where the recorded eye movements were slower than 80°/s and where saccades were shorter than 25 ms. Thereafter, in the remaining time periods where saccades were found, the 25 ms period (e.g., 5 samples) where the saccade velocity was highest during the saccade was determined and the average saccade velocity during this 25 ms period was calculated. If the subject made several saccades to achieve the target movement, the saccade with the highest peak saccade velocity and with the largest amplitude was selected.

The saccade amplitude was calculated as the distance moved from a start position where the eye movements began to exceed 80°/s velocity, until an end position i.e., where the velocity had decreased below this velocity. The saccade accuracy for each target movement was calculated as a quotient value in percent between the movement amplitude of the largest eye movement saccade (if several saccades were made), divided by the total movement amplitude of the visual target reference.

Finally, a value describing the general ratio between peak saccade velocity and saccade amplitude, commonly called main sequence [36,37], was calculated. This was done by first dividing peak saccade velocity by saccade amplitude for all individual saccade target amplitudes to give a quotient value for each saccade amplitude. Thereafter, the average quotient value for all saccade target amplitude quotients were calculated, which represents the general ratio.

#### Statistical analysis

Oculomotor performance was based on statistical analysis of rightward and leftward smooth pursuit and saccadic eye movements pooled together since no general differences were found in pair-wise Wilcoxon comparisons between values obtained from leftward and rightward directed smooth pursuits and saccades (average p-value = 0.521) [26,32].

The effects of DBS OFF and ON (denoted DBS in the tables) (1 d.f. : OFF or ON); the effects on smooth pursuit velocity (Target Velocity) (3 d.f. : 10°/s, 20°/s, 30°/s or 40°/s ), and the effects on saccade amplitude (Target Amplitude) (2 d.f. : 20°, 40° or 60°) and their respective interactions on the smooth pursuit and saccade parameters were analyzed using a repeated-measures GLM ANOVA (General Linear Model Analysis of Variance) test [38]. A repeated-measures GLM ANOVA analysis uses all available data from the assessments made to build a statistical model that shows how a parameter is affected by various factors and interactions between factors. In this study, the analysis include data from both DBS OFF and ON assessments and all Target Velocities/ Target Amplitudes respectively to determine whether the parameter is influenced by the main factors (DBS, Target Velocity/Target Amplitude) and interactions between the main factors (e.g., that DBS ON enhance more the ability to perform faster smooth pursuits than slower ones).

The Wilcoxon matched-pairs signed-rank test (Exact sig. 2-tailed) was used for post-hoc statistical evaluation of all parameter used, analyzing the differences between DBS states at the level of individual smooth pursuit velocities and saccade amplitudes [38].

A correlation analysis was performed using Spearman’s Rank Correlation test between UPDRS scores and smooth pursuit and saccade eye movements in DBS ON and OFF states. Non-parametric statistics were used in the statistical evaluation since not all data sets were normally distributed before or after logarithmic transformation. In the statistical analysis, p-values <0.05 were considered statistically significant [38].

## Results

### Smooth pursuit eye movements

#### Repeated-measures GLM analysis of smooth pursuit parameters

Generally, DBS ON significantly improved smooth pursuit gains (p = 0.005) and smooth pursuit velocity accuracies (p < 0.001) compared with DBS OFF (Table 2). Moreover, smooth pursuit gains were significantly higher for slower smooth pursuit target velocity movements (p < 0.001). Findings also show that it was easier for the PD patients to maintain correct smooth pursuit velocity within the velocity boundaries during faster smooth pursuit movements (p < 0.001). Furthermore, a significant interaction between DBS and target velocity (p = 0.034) for the gain parameter shows that DBS ON particularly improved the smooth pursuit gains values for the faster smooth pursuits. There was no significant interaction between DBS and target velocity for the smooth pursuit accuracy parameter.

**Table 2:** Repeated-measures GLM ANOVA analysis of smooth pursuit parameters

**Smooth pursuit parameters**	**Parameter values**			**p-values**		
	**Target velocities (°/s)**	**DBS OFF**	**DBS ON**	**DBS**	**Target velocity**	**DBS × Target velocity**
Gain	10	0.97 (0.03)	1.00 (0.01)	**0.005 (11.0)**	**<0.001 (31.5)**	**0.034 (5.4)**
	20	0.86 (0.04)	0.97 (0.01)			
	30	0.83 (0.05)	0.94 (0.02)			
	40	0.80 (0.04)	0.90 (0.02)			
Velocity Accuracy	10	31.7 (3.1)	39.0 (2.5)	**<0.001 (18.1)**	**<0.001 (35.0)**	0.188 (1.9)
	20	37.3 (3.7)	48.2 (3.1)			
	30	35.8 (3.9)	50.3 (3.5)			
	40	35.9 (3.3)	46.1 (2.5)			

Statistical evaluation of the smooth pursuit parameters (mean and SEM) using the repeated-measures GLM ANOVA method. Bold values indicate found significance (p < 0.05). The statistical F-values are presented within the parentheses after the p-values.

#### Post hoc analysis of smooth pursuit parameters

**Figure 1:**
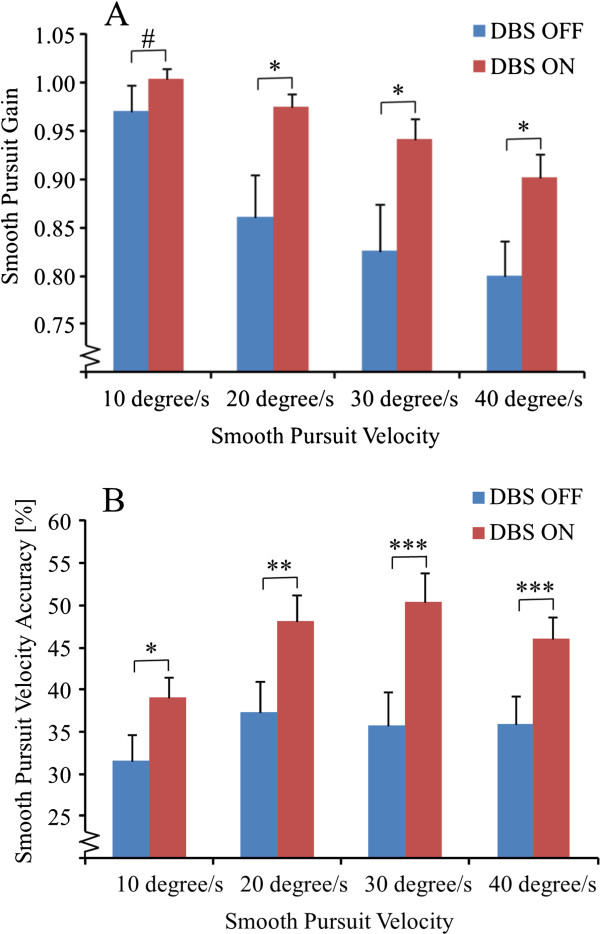
**A Gain values (mean and SEM) for four target velocities with DBS OFF and ON.** A value below 1.00 represent that the average smooth pursuit velocity was below the target velocity. **B**: Smooth pursuit velocity accuracy values (mean and SEM) with DBS OFF and ON. A value of 100 % represents that the eye movement velocity were always within the boundaries. (# denotes P < 0.1; * denotes P < 0.05; ** denotes P < 0.01 and *** denotes P < 0.001).

Post-hoc analysis of the smooth pursuit parameters at individual smooth pursuit velocities revealed that DBS ON significantly increased smooth pursuit gains with increasing target velocity compared with DBS OFF (Figure 1A). Smooth pursuit gains were significantly higher by 13% for the 20°/s and 40°/s velocities (p < 0.05) and by 14% for the 30°/s target velocity (p < 0.05) with DBS ON compared with DBS OFF. Smooth pursuit gain was not significantly different between DBS ON and DBS OFF for the 10°/s velocity, though a clear trend of increased smooth pursuit gain with DBS ON was observed (p = 0.074).

Smooth pursuit velocity accuracy was significantly increased with DBS ON compared with DBS OFF by 23% for 10°/s target velocity (p < 0.05); by 29% for 20°/s target velocity (p < 0.01); by 40% for 30°/s target velocity (p < 0.001) and by 28% for 40°/s target velocity (p < 0.001) (Figure 1B).

### Saccade eye movements

#### Repeated-measures GLM analysis of saccade parameters

Generally, the saccade amplitude accuracies were significantly higher (p = 0.007) with DBS ON compared with DBS OFF (Table 3). Moreover, a trend suggests that peak saccade velocity was increased by DBS ON though without reaching significant level (p = 0.087). Additionally, saccade velocities were significantly higher (p = 0.005) for larger saccade target amplitudes. Saccade amplitude accuracies were significantly poorer (p = 0.001) for the larger saccade target amplitudes. There were no significant interaction between DBS and target amplitude on the saccade parameters analyzed.

**Table 3:** Repeated-measures GLM ANOVA analysis of saccade parameters

**Saccade parameters**	**Parameter values**			**p-value**		
	**Target amplitudes (°)**	**DBS OFF**	**DBS ON**	**DBS**	**Target amplitude**	**DBS × Target amplitude**
Velocity	20	236 (16)	269 (18)	*0.087 (3.4)*	**0.005 (10.8)**	0.117 (2.8)
	40	263 (20)	288 (15)			
	60	313 (34)	332 (23)			
Amplitude Accuracy	20	53.4 (4.2)	65.8 (5.4)	**0.007 (10.2)**	**0.001 (17.0)**	0.575 (0.4)
	40	38.9 (4.4)	49.0 (2.8)			
	60	34.6 (5.2)	52.8 (5.3)			

Statistical evaluation of the saccade parameters (mean and SEM) using the repeated-measures GLM ANOVA method. Bold values indicate found significance (p < 0.05), italic values indicate trends of significance (p < 0.1). F-values are presented within the parentheses after the p-values.

#### Post hoc analysis of saccade parameters

**Figure 2:**
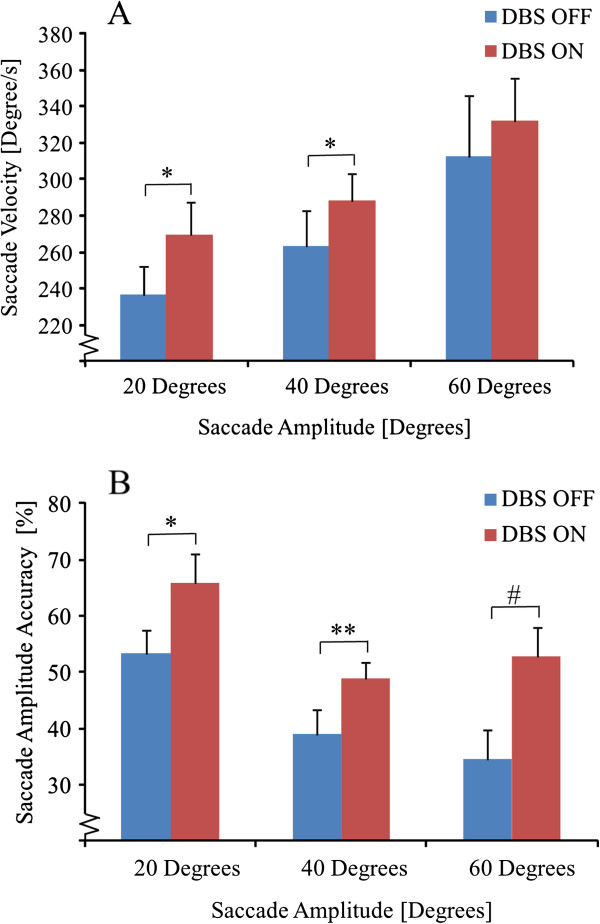
**A Saccade eye movement velocity (mean and SEM) for three saccade amplitudes with DBS OFF and ON. B**: Saccade amplitude accuracy in percentage (mean and SEM) with DBS OFF and ON. A value of 100 represents perfect saccade amplitude accuracy whereas values below 100 represent short saccades (hypometric) (# denotes P < 0.1; * denotes P < 0.05; ** denotes P < 0.01 and *** denotes P < 0.001).

Post-hoc analysis of the saccade parameters at individual saccade amplitudes revealed that saccade velocity was significantly increased with DBS ON compared with DBS OFF by 14% for the 20 degree saccade target amplitude (p < 0.05) and by 9% for the 40 degree saccade target amplitude (p < 0.05) (Figure 2A). Saccade velocity was not significantly different between DBS ON and DBS OFF for the 60 degree saccade target amplitude.

Saccade amplitude accuracy was significantly increased with DBS ON compared with DBS OFF by 23% for the 20 degree saccade target amplitude (p < 0.05) and by 26 % for the 40 degree saccade target amplitude (p < 0.01) (Figure 2B). Saccade amplitude accuracy was not significantly different between DBS ON and DBS OFF for the 60 degree saccade target amplitude, though a clear trend of increased saccade amplitude accuracy with DBS ON was observed (p = 0.065).

The saccade ratio between saccade velocity and saccade amplitude were on average 19.9 (standard error of mean (SEM) 0.8) with DBS OFF and 16.1 (SEM 0.7) with DBS ON (p < 0.001), the decrease in saccade ratio found for DBS ON was mainly due to increased saccade amplitudes.

### Smooth pursuit and saccade latencies

Average latency for saccades was 177 ms (SEM 12 ms) with DBS OFF and 156 ms (SEM 10 ms) with DBS ON. The saccade latency difference was not statistically significant between DBS ON and DBS OFF.

Average latency for smooth pursuits was 171 ms (SEM 14 ms) with DBS OFF and 218 ms (SEM 13 ms) with DBS ON. The smooth pursuit latency difference was not statistically significant between DBS ON and DBS OFF though a trend of increased smooth pursuit latency with DBS ON was observed (p = 0.090).

### Relationship between UPDRS scores and oculomotor parameters

The correlation analyses between UPDRS motor scores with DBS ON and smooth pursuit parameters with DBS ON revealed indications of relationships (Table 4A). The UPDRS scores with DBS ON were significantly correlated to smooth pursuit velocity accuracy at 10°/s target velocity with DBS ON (p < 0.001), the correlation coefficient values suggesting that those with higher UPDRS scores had poorer smooth pursuit velocity accuracy. The correlations also suggest trends of relationships between UPDRS score and smooth pursuit gain (p = 0.094) and smooth pursuit velocity accuracy (p = 0.060) at 20°/s target velocity.

**Table 4:** Correlation values between UPDRS motor scores and oculomotor parameters

**A: Smooth pursuit parameters**		**p-values (correlation coefficient)**	
	**Target velocities (°/s)**	**DBS OFF**	**DBS ON**
Gain	10	0.305 (−0.256)	0.131 (−0.369)
	20	0.135 (−0.366)	***0.094 (−0.406)***
	30	0.168 (−0.339)	0.527 (−0.160)
	40	0.243 (−0.310)	0.670 (−0.108)
Velocity Accuracy	10	0.150 (−0.354)	**<0.001 (−0.759)**
	20	0.767 (−0.075)	***0.060 (−0.452)***
	30	0.627 (−0.123)	0.532 (−0.158)
	40	0.466 ( 0.196)	0.521 (−0.162)
Latency		0.265 (−0.320)	0.828 ( 0.059)
**B: Saccade parameters**	p-values		
	Target amplitudes (°)	DBS OFF	DBS ON
Velocity	20	0.705 (−0.099)	***0.080 (−0.423)***
	40	0.313 ( 0.252)	***0.055 (−0.460)***
	60	0.539 ( 0.166)	0.338 (−0.248)
Amplitude Accuracy	20	0.518 (−0.168)	**0.048 (−0.473)**
	40	0.503 ( 0.169)	**0.048 (−0.473)**
	60	0.905 (−0.033)	0.811 (−0.063)
Latency		0.404 ( 0.217)	**0.009 (−0.614)**

Table 4A Correlation values between UPDRS motor scores and smooth pursuit parameters with DBS ON and DBS OFF. The correlation coefficient values are presented within the parentheses. Table 4B. Correlation values between UPDRS scores and saccade parameters with DBS ON and DBS OFF. Bold values indicate found significant correlation (p < 0.05), italic values indicate trends of correlation (p < 0.1). A negative correlation coefficient value suggests that PD subjects with higher UPDRS scores had poorer performance in the oculomotor parameter analyzed.

The correlation analysis between UPDRS scores with DBS ON and saccade parameters with DBS ON revealed several significant relationships (Table 4B). The UPDRS scores with DBS ON were significantly correlated to the saccade amplitude accuracy at 20° saccade target amplitude (p < 0.05) and saccade amplitude accuracy at 40° saccade target amplitude (p < 0.05) with DBS ON, the correlation coefficient values suggesting that those with higher UPDRS scores had poorer saccade amplitude accuracy. The UPDRS scores were significantly correlated to the saccade latency with DBS ON (p < 0.01). The correlations also suggest trends of relationships between UPDRS scores and the saccade velocity at 20° saccade target amplitude (p = 0.080) and saccade velocity at 40° saccade target amplitude (p = 0.055).

There were no significant correlations between UPDRS scores with DBS OFF and any of the smooth pursuit parameters or saccade parameters with DBS OFF.

## Discussion

### Smooth pursuit eye movements and deep brain stimulation

Although DBS in the STN is a highly effective therapeutic intervention for PD, its mechanism and effects on smooth pursuit eye movements have to date been rarely investigated [39]. One previous study from Pinkhardt and colleagues has investigated the effect of STN stimulation on smooth pursuit while the participants received anti-PD medication and found then no significant effect of DBS [29]. However, the present study reveals that when STN stimulation was applied alone after withdrawal of PD medication for 12 hours, DBS significantly improved smooth pursuit performance, both in terms of increasing smooth pursuit velocity gain but also by improving the ability to maintain a steady eye movement velocity without using supportive saccade disruptions or velocity deviations. The improvements made with DBS ON were more detectable at faster target velocities both in smooth pursuit gain and smooth pursuit velocity accuracy. Comparing the study by Pinkhardt and colleagues to the present study there were large differences, both methodological (i.e. different test situations) and between the participants (ours had longer disease duration, i.e., 16.6 versus 10.5 years). Discrepancies between study results may also be caused by differences in electrode locations and DBS settings, which are not always reported [22,24,25,28]. In order to gain an increased understanding of how STN stimulation affects oculomotor performance, it may be beneficiary if forthcoming studies report both electrode locations and DBS-parameter settings since these are of importance for the results. Hence, the recorded effectiveness of applied DBS is likely related to how strong the DBS stimulation is set to be and to what proportional degree the PD symptoms are suppressed by DBS stimulation or anti-PD medication respectively.

The observed, consistently positive, effects of STN stimulation on smooth pursuit performance contrasts with the lower coherency in the effects reported for PD medication. Some have reported that dopaminergic therapy improved smooth pursuit velocity gain [11,14], others have detected no difference [30], whereas some have even found that dopaminergic therapy worsened smooth pursuit velocity gain [31]. Hence, more research is motivated to investigate whether STN stimulation can provide a better stable improvement of smooth pursuit performance than PD medication or whether induced performance enhancements are associated with other factors such as state of PD progression.

The relationship between PD and smooth pursuit performance has been a subject of debate. There are a number of reports of decreased smooth pursuit performance in persons with PD [11,16,40,41]. Although abnormal smooth pursuit eye movements have been reported in about 75% of the PD patients [42], previous studies have been unable to conclude why this is as there appeared to be no direct ocular pursuit pathways traversing the BG [13]. However, new findings made by Yoshida and Tanaka [43] provide important physiological evidence for the existence of an oculomotor feedback circuit involving the putamen and globus pallidus, structures that have previously been associated in neurophysiological studies primarily with the somatomotor system. Although anatomical studies have previously showed projections from the thalamic nuclei, which receive input from the globus pallidus to the smooth pursuit subregion of the frontal eye field [44,45], the study by Yoshida and Tanaka showed a relationship between neural activity in the globus pallidus and the pursuit eye movements. Hence, rather than being controlled primarily by areas in extrastriate cortex specialized for processing visual motion, smooth pursuit eye movements involve an extended network of cortical areas, including pursuit-related region in the frontal eye fields and newly identified routes involving structures previously associated with the control of saccades, including the basal ganglia, the superior colliculus, and nuclei in the brain stem reticular formation [46]. Additionally, improvements in smooth pursuit with STN stimulation could be associated with immediate increases in attention brought about by re-activation of dopamine pathways [47]. Attention is a well-known actor in the control of smooth pursuit performance [32,33]. Interestingly, the STN and globus pallidum have direct connections to the attention network [47].

### Saccadic eye movements and deep brain stimulation

Saccade eye movements were clearly improved by STN stimulation alone, though mainly their amplitude accuracy. These improvements were clearer for smaller saccadic amplitudes (i.e., at 20 and 40 degrees), suggesting that STN stimulation improves the capacity to perform controlled small amplitude saccades more than the capacity to perform large amplitude saccades. One previous study did not detect any effect on saccades when the STN stimulation was turned ON, but their values were close to normal already with anti-PD medication and with the DBS turned OFF [29]. The improved saccadic performance found with DBS ON in the present study is in line with other reports [22-24,27]. Given, the vast improvements in saccadic performance observed in the present study as well as others, STN stimulation may re-establish direct signals involved in saccadic eye movements, such as to the superior colliculus. One strong possibility is that STN stimulation affects the substantia nigra reticulate-superior colliculi pathway since the striatum has a major outflow of signals via the substantia nigra pars reticulata to the superior colliculus [14]. In line with these findings, previous studies show that in untreated PD, poor smooth pursuit function triggers catch-up saccades [42] of small amplitude [48].

The saccade ratio shows that the changes in relationship between peak saccade velocity and saccade amplitude were markedly different in DBS OFF and ON states. As illustrated in Figure 2, although the peak saccade velocity was maintained fairly high, the saccade amplitude was strikingly lower with DBS OFF compared with DBS ON. Activation of DBS partly restored the peak saccade velocity versus saccade amplitude relationship from 19.9 (DBS OFF) to 16.1 (DBS ON), which can be compared with 12.8 in healthy subjects [33]. Our findings confirm previous observations by Rascol and colleagues [16] of abnormal relationships between saccade velocity and saccade amplitude in persons with PD. This study also revealed that STN stimulation can partly restore a more normal relationship by enhancing the ability to perform controlled larger saccade amplitudes.

### Saccade and smooth pursuit latency

Eye movements have sometimes been found hyper-reflexive in patients with PD as compared to healthy controls, possibly due to PD-induced changes in both the peripheral perceptual processing and in central executive mechanisms involving the BG [49]. Noteworthy, the average smooth pursuit latency value found with DBS ON (218 ms) was almost identical to that found in healthy subjects (221 ms) [33], whereas the latency with DBS OFF was 171 ms. A potential explanation for this could be that STN stimulation might suppress a hyperactive start of the smooth pursuit eye movements and thereby allow the visual target to be captured better at movement onset. However, it is interesting that patients with PD show impaired predictive smooth pursuit eye movements in the early stage [50], indicating multi-component dysfunction.

In our participants, STN stimulation also caused a small, but non-significant, shortening of saccadic latencies from 177 ms with DBS OFF to 156 ms with DBS ON, which is quite similar to that found in healthy subjects (153 ms) [33]. Similarly, Yugeta and colleagues found that STN stimulation improved saccade latency of memory guided and visually guided saccades [27].

### Relationship between UPDRS motor scores and oculomotor findings

The correlation analysis revealed that many of the oculomotor parameters were significantly related to the UPDRS motor scores (i.e. part III) or showed trends of such relationship. Saccadic latency reduction has previously been shown to correlate with improvements of motor symptoms with the STN stimulation turned ON [26]. This was however not shown in the study by Pinkhardt and colleagues [29] possibly because of the continued use of anti-PD medications. Lohnes and Earhart evaluated the effects of STN stimulation alone, and their UPDRS III scores correlated with the percentage improvement in saccade amplitude, but not with saccade velocity [24]. Baseline postural instability and gait scores of the UPDRS part III did however correlate with improvements in both saccade amplitude and velocity. Their findings may indicate that oculomotor performance in people with PD relates more to gait and balance problems than motor symptoms per se (i.e. as assessed by the UPDRS part III). This further highlights the importance of taking oculomotor performance into account while simultaneously investigating balance problems in PD.

In the present study, the statistical relationships found between smooth pursuit and saccade performance and the UPDRS motor scores were only present with DBS ON. To speculate, one factor that could substantially influence many scores assessed by UPDRS in DBS OFF state, without influencing oculomotor performance, is tremor. Notably, many subjects had clear tremor with DBS OFF but the STN stimulation almost completely eliminated this symptom in DBS ON in nearly all subjects assessed. Hence, the UPDRS scores may not always properly reflect the oculomotor functions in certain states, as illustrated in this study particularly with DBS OFF and without PD medication. This observation suggests that important functions, such as oculomotor performance, might have to be assessed separately in each state to properly provide detailed supplementary information to the general UPDRS motor scoring, congruent to other reports [22,23].

### Confounding factors

This study was performed on a limited number of subjects. The main reason for this was the restricted inclusion criteria applied to avoid interacting and confounding factors. For example, the subjects investigated were within a limited age range and were assessed within a limited time after DBS surgery. Possible confounding or interacting factors such as time after surgery, age at surgery, sickness duration and PD medication at the time of investigation were investigated using correlation analysis. In the material investigated, none of these factors were found to have a systematic influence on oculomotor performance, though such influences cannot and should not be excluded on general basis. Hence, this study was conducted on a well-defined group to ensure that the effects observed were most likely from STN stimulation alone. The statistical evaluation of the assessments revealed consistent effects among the subjects, evidencing that both saccadic and smooth pursuit eye movements were significantly improved by STN stimulation in persons with PD.

A factor difficult to account for is whether STN stimulation also influences attention and cognitive functions. The STN receives projections involved in emotional and cognitive activities from the anterior cingulate, inferior frontal cortex and medial and dorsolateral pre-frontal cortices [47], and an integrative function has been suggested [51]. Indeed, recent evidence suggests that DBS stimulation of the STN may have some involvement in modifying attentional cerebral networks [47]. Noteworthy, attention is also important for human motor control and for oculomotor performance [52,53]. Hence, one cannot rule out that improvements in attention and cognition partly account for the improvements recorded in oculomotor performance with STN stimulation. However, given the revealed relationships between several oculomotor parameters and UPDRS motor scores assessed with DBS ON, it is likely that not only is oculomotor function changed by any such attention enhancement induced by STN stimulation, but also motor functions assessed by the UPDRS.

### Clinical Implications

Properly functioning saccadic and smooth pursuit eye movements are vital because these functions bring visual objects or areas of interest into visual focus and keep moving objects in focus. Through these abilities, vision provides exteroceptive information that allows us to interact with a highly dynamic environment and supports feedforward motor control which helps us to anticipate change [54]. Hence, the improvements gained by STN stimulation on oculomotor functions are likely to have positive implications for patients’ ability to perform tasks that rely on visual motor control and visual feedback such as postural control [55] and reduce side-effects from otherwise present poor oculomotor control like visual distortions and dizziness.

Currently, many evaluations concerning persons with PD (e.g., effectiveness of therapeutic interventions including DBS and progression of the disease) are based on conventional observer-dependent rating scales such as the UPDRS. This study illustrates that analysis of oculomotor functions might provide an additional information source when evaluating the effects of interventions like STN stimulation. The largely automatic procedures used when performing the assessments and analyzing the data ensures that the patients can be objectively assessed repeatedly using a high resolution evaluation scale.

### Methodological considerations

The positive influence of STN stimulation on the ability to perform accurate saccadic and smooth pursuit eye movements was most clearly illustrated by the novel smooth pursuit velocity accuracy analysis and the saccadic ratio parameter, the latter commonly called main sequence [36,37].

The novel smooth pursuit velocity accuracy parameter provides information about to what degree a steady smooth pursuit velocity can be maintained without supportive saccades or velocity inaccuracies beyond a 20% velocity error limit. As evidenced in two independent studies [32,33] and the phenomena itself previously described in other studies [56,57], one of the first signs of an affected oculomotor function might be an inability to maintain steady and accurate control of smooth pursuit movements within acceptable velocity boundaries over longer periods of time. Furthermore, Armstrong suggests that this method might prove successful for evaluating PD [58]. In the present study and in previous studies [11,16,40,41], the traditional smooth pursuit gain analysis also provided significant statistical evidence for an altered smooth pursuit function, but not with the same high statistical sensitivity as the velocity accuracy parameter. Hence, analysis of smooth pursuit velocity accuracy may provide better means to detect oculomotor deficits at an earlier stage in a number of disorders and in persons with PD where other functional parameters may not.

The saccade ratio parameter illustrates whether there is an imbalance between saccade velocity and the saccade amplitude. Previous reports have shown a relatively fixed relationship between the saccade amplitude and saccade velocity up to about 15–20 degrees amplitude. However, above these saccade amplitudes the relationship changes in a non-linear manner [32,36,37]. Recent reports have shown that this relationship can be changed by various factors with central effects such as alcohol intoxication and sleep deprivation [32,33]. Such saccade relationship changes between saccade velocity and the saccade amplitude were also detected in persons with PD when using the saccade ratio parameter to assess the effects of DBS in the STN.

The possible relationship between UPDRS scores and oculomotor findings were investigated by performing correlations to each of the oculomotor parameters. One should be aware of that multiple analyses can increase the risk of statistical Type 1 errors but also that the Bonferroni correcting factor under some conditions could increase the risk of making Type II errors [59]. Specifically, it is difficult to present multiple systematic relationships in the p-value range of <0.05 to >0.01 without all of them being down-ranked to insignificant level by Bonferroni. Contradictory, if publishing one finding at a time, as singled-out observations, then they are individually regarded as significant evidence for the hypothesis. Moreover, as described by Perneger [59], if Bonferroni was applied in clinical practice the effects would be absurd. Hence, it is important that systematic investigations can be published, i.e., both significant and insignificant findings, because both kinds of findings are often of direct clinical relevance. Accordingly, we present the complete statistical correlation analysis of all oculomotor parameters so the systematic patterns are displayed, though we do so with a reminder of being aware of the problems associated with Type I and Type II errors.

#### Conclusions

STN stimulation from DBS alone significantly improves smooth pursuit and saccade performance in patients with PD. The improved oculomotor functions with DBS in the STN may have positive implications for patients’ ability to perform tasks that rely on visual motor control and visual feedback (such as postural control) and reduce side-effects from poor visual control like visual distortions and dizziness. The findings further indicate a critical but complex role of the basal ganglia and STN on oculomotor functions. Moreover, the new oculomotor analysis methods used provide a sensitive vehicle to detect functional enhancements produced by STN stimulation from DBS alone. These methods could be useful in detecting subtle pathological changes from PD. The largely automatic procedures used when performing the assessments and analyzing the data ensures that the patients can be objectively assessed repeatedly using a high resolution evaluation scale.

## Competing interests

The authors declare that they have no competing interests.

## Authors’ contributions

MN participated in the design of the study, recruited the patients, managed acquisition of data, assisted in data analysis and drafted the manuscript. MP assisted in data analysis and drafted the manuscript. SR and MM participated in the project organization, design and helped draft the manuscript. PAF participated in the design of the study, collecting data, performed data analysis and drafted the manuscript. All authors read and approved the final manuscript.
